# DAMPs and NETs in Thyroid Diseases: Biological, Diagnostic, and Translational Perspectives

**DOI:** 10.3390/cancers18213469

**Published:** 2026-10-27

**Authors:** Kamil Waczyński, Barbara Choromańska, Mateusz Maciejczyk, Marta Lewoc-Magnuszewska, Alan Tkaczuk, Julia Grymuza, Jacek Dadan, Piotr Myśliwiec

**Affiliations:** 1 1st Department of General and Endocrine Surgery, Medical University of Bialystok, 24a M. Sklodowskiej-Curie Street, 15-276 Bialystok, Poland; barbara.choromanska@umb.edu.pl (B.C.); marta.lewoc@sd.umb.edu.pl (M.L.-M.); alan.tkaczuk@sd.umb.edu.pl (A.T.); julia.grymuza@sd.umb.edu.pl (J.G.); jacek.dadan@umb.edu.pl (J.D.); 2 Department of Hygiene, Epidemiology and Environmental Health, Medical University of Bialystok 2c A. Mickiewicza, 15-369 Bialystok, Poland; mateusz.maciejczyk@umb.edu.pl; 3 Department of Minimally Invasive Surgery, Medical University of Bialystok, 24a M. Sklodowskiej-Curie Street, 15-276 Bialystok, Poland; piotr.mysliwiec@umb.edu.pl

**Keywords:** thyroid carcinoma, neutrophil extracellular traps, damage-associated molecular patterns, biomarkers, liquid biopsy, innate immunity

## Abstract

Thyroid diseases encompass a broad spectrum of autoimmune, inflammatory, and neoplastic disorders, yet current diagnostic approaches provide limited insight into innate immune activation and tumor-associated inflammatory remodeling. Damage-associated molecular patterns (DAMPs) and neutrophil extracellular traps (NETs) have emerged as interconnected mediators of sterile inflammation with potential relevance to thyroid carcinogenesis, disease progression, and biomarker development. This narrative review synthesized literature identified through PubMed/MEDLINE and Scopus to evaluate the biological, diagnostic, and translational significance of DAMP- and NET-associated pathways in thyroid diseases, with particular emphasis on thyroid cancer. DAMPs such as high-mobility group box 1 (HMGB1), S100A8/A9, serum amyloid A, heat shock proteins, and extracellular nucleic acids may sustain inflammatory signaling through pattern-recognition receptors and contribute to tumor proliferation, angiogenesis, immune modulation, and therapeutic resistance. In parallel, circulating analytes associated with NET biology—including nonspecific cell-free DNA (cfDNA) and nucleosomes, pathway-associated citrullinated histone H3 (CitH3), and composite myeloperoxidase–DNA (MPO–DNA) complexes—have shown associations with thyroid malignancy, dedifferentiated disease, metastatic presentation, and greater disease severity. Experimental data further suggest that aggressive thyroid cancer cells, particularly anaplastic thyroid carcinoma, can induce extracellular trap formation and exploit neutrophil-derived signals to support tumor growth. However, limited disease specificity, assay heterogeneity, and the predominance of small or exploratory cohorts currently restrict clinical implementation. Future strategies should prioritize standardized, prospective validation of multimarker panels integrating DAMP- and NET-related analytes with imaging, cytology, molecular diagnostics, and clinicopathological risk stratification to determine whether they provide incremental value for diagnosis, prognostication, and longitudinal monitoring in thyroid cancer.

## Simple Summary

Thyroid diseases include inflammatory, autoimmune, and cancerous conditions, but routine tests do not fully show how innate immune responses contribute to disease activity. In this narrative review, we examined published evidence on two related processes: damage-associated molecular patterns, which are released by stressed or injured cells, and neutrophil extracellular traps, which are produced by activated neutrophils, with particular attention to evidence obtained directly in thyroid diseases. Current evidence suggests that these markers may help reflect inflammation, tumor aggressiveness, metastatic disease, and treatment resistance in thyroid cancer. However, available studies are often small and use different laboratory methods. Future research should validate standardized combinations of these biomarkers together with imaging, cytology, molecular testing, and clinical risk assessment before they can be used routinely in patient care.

## 1. Background

Thyroid disorders, encompassing functional abnormalities, structural lesions, autoimmune diseases, and thyroid malignancies, represent an increasingly important health concern. Their growing prevalence and detection rates pose a substantial challenge to healthcare systems worldwide. Thyroid-related conditions are estimated to affect even one in five individuals [1]. These disorders show a marked female predominance, although the magnitude of the sex disparity varies according to the specific condition. Their prevalence also increases with age. Among adults aged 60 years or older, the estimated prevalence of thyroid dysfunction is 15.5%, reaching 21.5% in women and 7.2% in men, while approximately 3.2% of cases remain undiagnosed [2]. The global burden of thyroid disease also includes a steadily increasing number of thyroid cancer diagnoses. According to estimates for 2022, approximately 821,000 new cases of thyroid cancer and 47,500 related deaths occurred worldwide. The age-standardized incidence rate was almost threefold higher in women than in men, at 13.6 versus 4.6 cases per 100,000 population, respectively [3].

The most common thyroid pathologies include hypothyroidism, hyperthyroidism, nodular goiter, and thyroid tumors. The most prevalent autoimmune thyroid diseases are Hashimoto’s thyroiditis (HT) and Graves’ disease (GD). The prevalence of hypothyroidism in the general population may reach approximately 5%, while the prevalence of undiagnosed hypothyroidism, including overt and mild forms, has also been estimated at around 5% [4]. More than 99% of cases are classified as primary hypothyroidism. HT, the most common cause of primary hypothyroidism worldwide, is characterized by T-cell-mediated destruction of the thyroid parenchyma. This progressive process leads to a chronic hypometabolic state that may manifest clinically as hyperlipidemia, bradycardia, increased cardiovascular risk, and depressive symptoms. HT has also been associated with an increased risk of papillary thyroid carcinoma (PTC). Hyperthyroidism is estimated to affect approximately 0.8% of the European population. Its prevalence increases with age and is higher in women than in men [5]. GD is the most common cause of hyperthyroidism and results from stimulation of the thyroid-stimulating hormone (TSH) receptor by thyrotropin receptor antibodies (TRAb). This leads to hypermetabolism, increased sympathetic activity and may result in clinically significant complications, including heart failure, osteoporosis, and characteristic ocular manifestations such as proptosis.

Thyroid cancer accounts for approximately 4% of all cancers in women, and its incidence continues to increase [6]. The average incidence rate in Europe is 10.2 per 100,000 person-years, with rates of 15.4 per 100,000 person-years in women and 5.0 per 100,000 person-years in men [7]. Thyroid malignancies comprise several histological subtypes with markedly different incidence rates, biological behavior, and prognoses.

Papillary thyroid carcinoma (PTC), the most common histological subtype, accounts for approximately 80–85% of all thyroid cancers. It generally has the most favorable prognosis and typically presents as a slow-growing and highly treatable malignancy. Follicular thyroid carcinoma (FTC) is the second most common subtype and accounts for approximately 10–15% of cases. PTC and FTC are collectively classified as differentiated thyroid carcinoma (DTC) because they retain morphological and functional characteristics of normal thyroid follicular cells and may remain responsive to radioactive iodine therapy. Less common thyroid malignancies include medullary thyroid carcinoma (MTC), which originates from parafollicular C cells and accounts for approximately 3–4% of thyroid cancers. MTC may occur sporadically or as part of hereditary syndromes, including multiple endocrine neoplasia type 2 (MEN2). Anaplastic thyroid carcinoma (ATC), an undifferentiated malignancy of follicular-cell origin, is the rarest and the most aggressive subtype, accounting for approximately 1–2% of thyroid cancers. ATC is associated with rapid disease progression, resistance to conventional therapies, and a very poor prognosis. The present review focuses primarily on follicular-cell-derived thyroid carcinomas, particularly PTC, FTC, and ATC, for which the available DAMP- and NET-related evidence is most directly relevant. MTC and other rare histological subtypes are not discussed in detail because thyroid-specific evidence linking DAMP- and NET-associated mechanisms to these entities remains insufficient for a focused synthesis.

### 1.1. Pathogenesis of Thyroid Disorders

The development of autoimmune thyroid disease is influenced by a complex interplay of genetic, environmental, hormonal, and immunological factors. Established or proposed risk factors for HT and GD include female sex, genetic susceptibility, a family history of autoimmune thyroid disorders, excessive or deficient iodine intake, smoking, infections, psychological stress, pregnancy and the postpartum period, exposure to ionizing radiation, and the use of selected medications, including amiodarone, lithium, interferon-α, and immune checkpoint inhibitors.

Risk factors for thyroid cancer vary according to histological subtype and include exposure to ionizing radiation, particularly during childhood, a family history of thyroid malignancy, hereditary cancer syndromes, pre-existing thyroid nodules or goiter, iodine imbalance, obesity, and increasing age. Additional subtype-specific risk factors include germline RET proto-oncogene mutations in MTC and prolonged TSH stimulation in DTC.

Emerging evidence suggests that damage-associated molecular patterns (DAMPs) and neutrophil extracellular traps (NETs) contribute to sterile inflammation and sustained immune activation within the thyroid microenvironment. Initially described as an antimicrobial defense mechanism, NETs are now recognized as important mediators of autoimmunity and cancer-associated inflammation. Excessive NET formation or impaired NET clearance may promote autoantigen exposure, cytokine release, plasmacytoid dendritic cell activation, and autoantibody production, thereby perpetuating local inflammatory responses in autoimmune thyroid diseases [8,9,10,11,12].

Beyond autoimmunity, thyroid cancer cells may modulate neutrophil activity and promote NET formation within the tumor microenvironment [13,14]. Experimental evidence indicates that NET-associated components may facilitate tumor cell proliferation, migration, pre-metastatic niche formation, and inflammatory remodeling, particularly in aggressive thyroid cancer phenotypes [15,16]. DAMP release and NET formation are mechanistically interconnected, as NET-associated molecules may themselves act as secondary DAMPs and further amplify inflammatory signaling. Although evidence regarding these mechanisms in thyroid disorders remains limited, their involvement in autoimmunity, chronic inflammation, and tumor biology suggests potential diagnostic and therapeutic relevance [17].

### 1.2. Diagnosis of Thyroid Diseases

The diagnostic evaluation of thyroid disorders integrates clinical assessment with biochemical, serological, imaging, and cytological findings. The diagnostic pathway depends on whether the predominant clinical problem involves functional or autoimmune disease, thyroid nodules, or suspected malignancy [18,19]. The evaluation of non-neoplastic thyroid disorders is primarily based on serum TSH, free thyroxine (FT4), and, when indicated, free triiodothyronine (FT3). These measurements enable the identification of overt and subclinical hypothyroidism or hyperthyroidism [19,20]. Autoantibody testing complements hormonal assessment: anti-thyroid peroxidase antibodies (anti-TPO) and anti-thyroglobulin antibodies (anti-Tg) support the diagnosis of HT, whereas TRAb are particularly relevant in GD [21,22,23]. Thyroid ultrasonography provides information on gland volume, echogenicity, vascularity, structural heterogeneity, and focal lesions [18,23]. Scintigraphy may be useful in selected patients, particularly for differentiating increased thyroid hormone synthesis from destructive thyroiditis or identifying autonomously functioning nodules [24]. However, these methods primarily characterize thyroid function, autoimmunity, and structural abnormalities rather than the intensity of underlying innate immune activation.

In patients with thyroid nodules or suspected malignancy, high-resolution ultrasonography is the principal imaging method used to identify features associated with an increased risk of cancer. Ultrasound-based risk-stratification systems guide the selection of nodules requiring fine-needle aspiration biopsy (FNAB) [18], while cytological findings are commonly classified according to the Bethesda System for Reporting Thyroid Cytopathology [25]. FNAB effectively distinguishes many benign from malignant lesions, although indeterminate cytological findings remain a major diagnostic challenge. Molecular testing for selected genetic alterations may improve risk assessment in such cases, but its availability and diagnostic performance vary [26]. Following a diagnosis of thyroid cancer, imaging, histopathological evaluation, and disease-specific biochemical markers are used for staging, risk stratification, and surveillance [27].

Despite significant advances in endocrinological diagnostics, the clinical assessment of thyroid diseases continues to rely primarily on hormonal measurements, including TSH, FT4, and FT3; autoantibody profiling with anti-TPO, anti-Tg, and TRAb; ultrasonography; and FNAB in nodular disease [18,21]. Although these tools are effective for diagnosis and functional evaluation, they provide limited insight into early inflammatory activation, the risk of disease progression, or tumor aggressiveness [28]. Moreover, reliable biomarkers capable of differentiating benign inflammatory nodules from malignant lesions, predicting autoimmune exacerbations, or reliably predicting malignant behavior remain insufficiently established [28]. Especially, distinguishing a benign follicular adenoma from a malignant follicular carcinoma remains a particular challenge. This diagnostic gap may potentially be addressed by molecular markers reflecting innate immune activation [14,29].

Given the central role of sterile inflammation in both autoimmunity and tumorigenesis, DAMPs and NET-associated components have emerged as promising candidates for diagnostic and prognostic biomarkers [30]. Their measurable presence in serum or tissue samples may reflect ongoing cellular stress, immune activation, or tumor-associated inflammatory remodeling [14]. It should be emphasized that direct thyroid-specific evidence remains limited for several DAMP- and NET-associated mechanisms. Accordingly, throughout this review, findings demonstrated directly in human thyroid disease or thyroid-specific experimental models are distinguished from mechanistic concepts extrapolated from broader oncological, inflammatory, or autoimmune settings.

The distinctive contribution of the present review is the integrated evaluation of DAMP- and NET-associated mechanisms across inflammatory and autoimmune thyroid diseases and thyroid cancer, while explicitly separating human thyroid-disease evidence, human thyroid-cancer evidence, thyroid-specific preclinical evidence, and evidence extrapolated from broader oncology and immunology. In addition, we critically distinguish mechanistic plausibility from demonstrated diagnostic or prognostic performance, evaluate assay-specific limitations, and summarize quantitative human clinical evidence in a dedicated clinical-evidence table.

### 1.3. Review Methodology

This article was conducted as a narrative review. A literature search was performed using PubMed/MEDLINE and Scopus to identify studies addressing damage-associated molecular patterns (DAMPs), neutrophil extracellular traps (NETs), innate immune activation, and their diagnostic, prognostic, and mechanistic relevance in thyroid diseases, with particular emphasis on thyroid cancer. The literature search was initiated on 15 January 2026 and last updated on 30 August 2026.

The search strategy combined thyroid disease-related terms, including “thyroid cancer”, “papillary thyroid carcinoma”, “anaplastic thyroid carcinoma”, “Hashimoto’s thyroiditis”, and “thyroid autoimmunity”, with biomarker- and mechanism-related terms, including “damage-associated molecular patterns”, “DAMPs”, “HMGB1”, “S100A8/A9”, “calprotectin”, “serum amyloid A”, “heat shock proteins”, “cell-free DNA”, “neutrophil extracellular traps”, “NETs”, “NETosis”, “citrullinated histone H3”, “MPO–DNA”, and “mitochondrial DNA”. Boolean operators AND and OR were used to combine disease- and biomarker-related terms.

English-language original articles, clinical studies, translational and experimental studies, systematic reviews, meta-analyses, and relevant authoritative reviews were considered for inclusion. Studies were selected based on their relevance to DAMP- or NET-related mechanisms, biomarker expression, diagnostic or prognostic associations, or therapeutic implications in thyroid diseases. Priority was given to studies involving human thyroid tissue, blood samples, thyroid cancer cell models, or thyroid-specific in vivo models.

Studies without relevance to DAMP/NET biology or thyroid diseases, conference abstracts lacking sufficient methodological information, and non-English-language publications were excluded. Because direct thyroid-specific evidence remains limited for several mechanisms, selected studies from broader oncology, immunology, and inflammation research were included to provide mechanistic context. These findings were explicitly distinguished from thyroid-specific clinical and experimental evidence.

Reference lists of relevant reviews and key primary studies were additionally screened to identify potentially eligible publications. Study selection was based on relevance to the predefined scope of this narrative review rather than on a formal systematic-review protocol. No quantitative meta-analysis or formal risk-of-bias assessment was performed. Consequently, the narrative approach introduces an inherent risk of selection and interpretation bias, and the evidence presented should not be considered exhaustive in the manner of a systematic review.

## 2. Inflammatory and Innate Immune Mechanisms in Thyroid Diseases

### 2.1. Sterile Inflammation

Sterile inflammation is an inflammatory response initiated in the absence of infectious pathogens. Unlike pathogen-driven inflammation, which is triggered by pathogen-associated molecular patterns (PAMPs), sterile inflammation is mediated predominantly by endogenous danger signals released during cellular stress, injury, or death. These DAMPs activate innate immune pathways through pattern-recognition receptors (PRRs), initiating and amplifying local inflammatory responses [31].

In the thyroid gland, sterile inflammation is particularly relevant to autoimmune thyroid diseases, including HT and GD. Progressive thyrocyte injury and cellular stress may promote the release or aberrant exposure of intracellular danger signals, including high mobility group box 1 (HMGB1), heat shock proteins, and mitochondrial DNA [32,33,34]. These molecules may sustain immune-cell recruitment, cytokine production, and chronic inflammatory signaling, thereby contributing to structural remodeling and progressive thyroid dysfunction [32,33].

Sterile inflammation has also been implicated in thyroid tumorigenesis. Persistent exposure to endogenous inflammatory mediators may promote oxidative stress, genomic instability, epigenetic alterations, and the development of a pro-tumorigenic microenvironment. Owing to its high vascularization and intense oxidative metabolism associated with thyroid hormone synthesis, the thyroid gland may be particularly susceptible to inflammation-related cellular stress and subsequent neoplastic transformation [35].

### 2.2. Pattern Recognition Receptors (PRRs)

PRRs are germline-encoded sensors of innate immunity responsible for detecting both PAMPs and DAMPs [36]. Major PRR families include Toll-like receptors (TLRs), nucleotide-binding oligomerization domain-like receptors (NLRs), and retinoic acid-inducible gene I-like receptors (RLRs), while the receptor for advanced glycation end products (RAGE) functions as a multiligand pattern-recognition receptor involved particularly in DAMP sensing [37].

In thyroid pathology, TLRs particularly TLR2, TLR3, TLR4, and TLR9 have been detected on thyrocytes and infiltrating immune cells [38]. Activation of these receptors induces downstream signaling, culminating in activation of nuclear factor kappa B (NF-κB) and the production of pro-inflammatory cytokines, including interleukin-6 (IL-6), tumor necrosis factor-α (TNF-α), and interferon-β (IFN-β). Persistent or dysregulated PRR signaling may enhance antigen presentation, immune-cell recruitment, and adaptive immune activation, thereby contributing to the amplification of autoimmune responses [36].

Beyond autoimmunity, PRR signaling has been implicated in thyroid tumorigenesis. Aberrant TLR4 overexpression has been demonstrated in PTC, including increased expression in lymph-node metastases, while functional TLR4 signaling activates NF-κB in PTC cell models; in BRAF V600E-positive tumors, high TLR4 expression was also associated with shorter disease-free survival [39]. These findings suggest that PRR activation represents a shared mechanistic axis linking innate immunity, chronic inflammation, and thyroid malignancy.

### 2.3. Chronic Inflammation and Tumorigenesis

Chronic inflammation is widely recognized as a hallmark of cancer. Persistent inflammatory signaling promotes cellular proliferation, inhibits apoptosis, enhances angiogenesis, and facilitates epithelial–mesenchymal transition (EMT). These processes are mediated by cytokines, including IL-6 and TNF-α; transcription factors, including NF-κB and signal transducer and activator of transcription 3 (STAT3); and reactive oxygen species (ROS) [31,35].

In the thyroid gland, chronic autoimmune inflammation, particularly in HT, has been proposed as a potential contributor to malignant transformation [40,41]. Continuous lymphocytic infiltration and cytokine release may induce DNA damage and alter the local microenvironment [41]. Furthermore, oxidative stress generated during inflammatory processes may promote DNA damage and genomic instability, while alterations in BRAF- and RAS-related signaling pathways are frequently observed in DTC [41,42].

Inflammatory mediators may also modulate tumor immune-evasion mechanisms by altering immune-cell polarization and promoting an immunosuppressive niche. Thus, chronic inflammation may represent not merely a bystander phenomenon but a potential driver of thyroid carcinogenesis [41,43].

### 2.4. The Link Between Hashimoto’s Thyroiditis and Papillary Thyroid Carcinoma

The association between HT and PTC remains controversial. Histopathological and epidemiological studies have reported a higher prevalence of PTC among patients with HT, although the strength of this association varies according to study design and diagnostic method [44]. In a meta-analysis including 27 studies and 76,281 patients, HT was found to be associated with an increased likelihood of PTC, with a pooled odds ratio of 2.12 [45]. However, the association was considerably stronger in thyroidectomy-based studies than in FNAB-based cohorts, suggesting that referral and selection bias may influence the observed risk [45].

Several mechanisms may contribute to the coexistence of HT and PTC. Persistent autoimmune inflammation may promote oxidative stress, repeated thyrocyte injury, cytokine release, and compensatory cellular proliferation. Increased TSH stimulation in patients with hypothyroidism and shared molecular alterations, including RET/PTC rearrangements, have also been proposed as potential links between chronic thyroid inflammation and carcinogenesis [41].

Despite the increased prevalence of PTC among patients with HT, tumors arising in this setting may demonstrate a more favorable clinicopathological profile, including lower rates of extrathyroidal extension and lymph-node metastasis [46,47]. This observation may reflect enhanced local immune surveillance [48]. Overall, current evidence supports an association between HT and PTC but does not establish a direct causal relationship [47,48].

### 2.5. Tumor Microenvironment in Thyroid Cancer

The tumor microenvironment (TME) in thyroid cancer comprises immune cells, including macrophages, neutrophils, and lymphocytes; fibroblasts; endothelial cells; extracellular matrix components; and soluble mediators. This dynamic ecosystem influences tumor growth, invasion, and therapeutic response [49,50].

In PTC, increased infiltration by tumor-associated macrophages (TAMs) has been found to correlate with advanced disease stage and poorer prognosis [51]. Neutrophils and their secretory products may contribute to extracellular matrix remodeling and angiogenesis [14,50]. Pro-inflammatory cytokines and chemokines further shape immune-cell recruitment and polarization [52].

**Figure 1:**
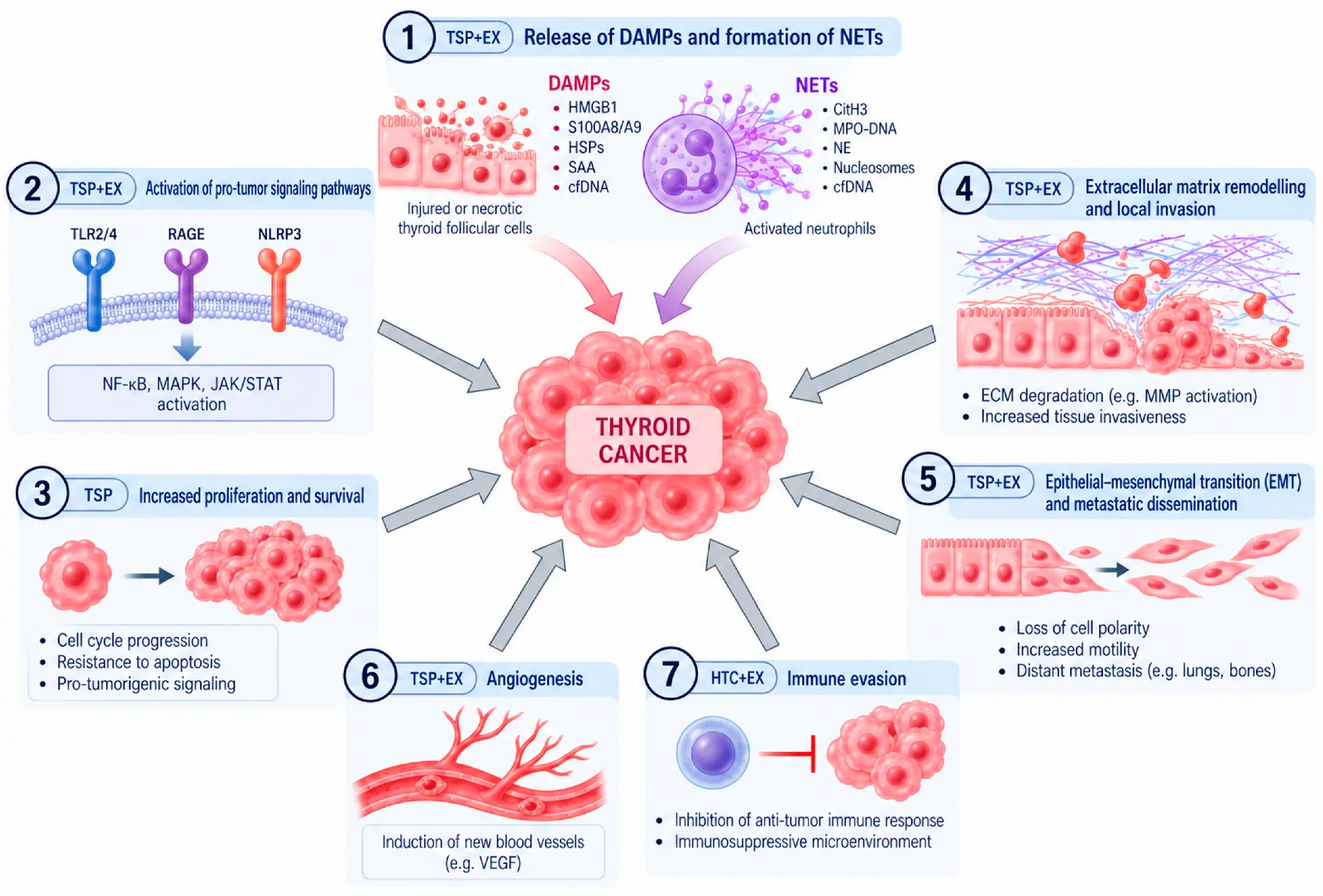
Potential impact of damage-associated molecular patterns (DAMPs) and neutrophil extracellular traps (NETs) on thyroid cancer. DAMP and NET release (1): Cellular stress and tumor-associated inflammation promote DAMP release and NET formation, amplifying inflammatory signaling. Pro-tumor signaling (2): DAMPs activate pattern-recognition receptors and downstream inflammatory pathways. Proliferation and survival (3): DAMP- and NET-associated signaling may enhance tumor-cell proliferation and resistance to apoptosis. Extracellular matrix remodeling and local invasion (4): NET-associated proteases and inflammatory mediators promote extracellular matrix remodeling and tumor invasiveness. EMT and metastatic dissemination (5): Persistent inflammatory signaling and NETs may facilitate EMT, tumor-cell migration, and metastatic progression. Angiogenesis (6): DAMP- and neutrophil-associated mediators promote tumor vascularization. Immune evasion (7): DAMPs, NETs, and activated neutrophils may contribute to an immunosuppressive tumor microenvironment and impaired antitumor immunity. Evidence categories follow those used throughout the manuscript: HTD, human thyroid-disease evidence; HTC, human thyroid-cancer evidence; TSP, thyroid-specific preclinical evidence; and EX, mechanistic evidence extrapolated from other malignancies or general cancer/immunology research. Because Figure 1 focuses specifically on thyroid cancer, HTD is not assigned to the pathways shown. Pathways 1 and 2 are supported by thyroid-specific experimental observations together with broader mechanistic evidence (TSP + EX); pathway 3 has direct thyroid-specific experimental support (TSP); pathways 4–6 are supported by selected thyroid-specific observations but also rely on mechanistic extrapolation from other cancers (TSP + EX); and pathway 7 combines human thyroid-cancer evidence on immunosuppressive neutrophil phenotypes with broader evidence on NET/DAMP-mediated immune evasion (HTC + EX). Additional abbreviations: HMGB1, high-mobility group box 1; S100A8/A9, S100 calcium-binding proteins A8 and A9; HSPs, heat shock proteins; SAA, serum amyloid A; cfDNA, cell-free DNA; DNA, deoxyribonucleic acid; CitH3, citrullinated histone H3; MPO–DNA, myeloperoxidase–DNA complexes; NE, neutrophil elastase; TLR2/4, Toll-like receptors 2 and 4; RAGE, receptor for advanced glycation end products; NLRP3, NLR family pyrin domain-containing 3; NF-κB, nuclear factor kappa B; MAPK, mitogen-activated protein kinase; JAK/STAT, Janus kinase/signal transducer and activator of transcription; ECM, extracellular matrix; MMP, matrix metalloproteinase; EMT, epithelial–mesenchymal transition; VEGF, vascular endothelial growth factor.

Emerging evidence indicates that innate immune mediators, including DAMPs and potentially NET-associated components, may participate in remodeling the TME and thereby influence tumor behavior. Understanding these interactions is essential for evaluating their diagnostic and prognostic utility [14,17]. The potential impact of DAMPs and NETs on thyroid cancer is summarized in Figure 1.

## 3. Damage-Associated Molecular Patterns

### 3.1. Molecular Classification of DAMPs

DAMPs are a heterogeneous group of endogenous molecules released or exposed following cellular stress, injury, necrosis, or dysregulation. Under physiological conditions, many of these molecules remain intracellular and immunologically silent; however, upon cellular damage, they enter the extracellular environment and activate innate immune responses. DAMPs include nuclear molecules, such as high mobility group box 1 (HMGB1), histones, DNA, and RNA; cytoplasmic and mitochondrial components, including extracellular ATP and mitochondrial DNA; stress-response proteins, such as heat shock proteins; inflammatory proteins, including S100A8/A9 and serum amyloid A; and extracellular matrix-associated molecules, such as decorin. Uromodulin has also been identified as a tissue-derived DAMP in selected pathological settings [53].

Extracellular DAMPs are recognized by PRRs, including TLRs, RAGE, and NLRs, leading to NF-κB activation, cytokine release, and amplification of inflammatory signaling [35,36]. In autoimmune thyroid diseases, persistent DAMP signaling from damaged thyrocytes may sustain inflammation, enhance antigen presentation, and facilitate adaptive autoimmune responses [32]. In thyroid cancer, DAMP-associated pathways may additionally contribute to tumor proliferation, angiogenesis, metastatic progression, and remodeling of the tumor immune microenvironment [14,54].

### 3.2. DAMPs in Oncogenesis

The mechanisms summarized in this section are largely derived from the broader oncology literature and should not be interpreted as having been uniformly demonstrated in thyroid cancer. Thyroid-specific evidence is discussed separately in Section 3.3.

DAMPs exert dual and context-dependent effects in oncology. They are increasingly recognized as important mediators linking chronic inflammation with tumor initiation, progression, and metastatic dissemination. Although acute inflammatory responses may exert antitumor effects, persistent DAMP-mediated signaling can promote a protumorigenic microenvironment characterized by chronic cytokine production, immune dysregulation, oxidative stress, angiogenesis, and extracellular matrix remodeling [35].

Tumor cells, stromal cells, necrotic tissue, and infiltrating immune cells may all serve as sources of DAMPs within the TME. Molecules such as HMGB1, S100 proteins, extracellular ATP, mitochondrial DNA, heat shock proteins, and extracellular nucleic acids are released during cellular stress, necrosis, hypoxia, chemotherapy-induced injury, or rapid tumor proliferation. These endogenous danger signals activate PRRs, including TLRs, RAGE, and inflammasome-associated sensors, leading to sustained inflammatory signaling [55,56].

DAMP-mediated signaling can engage NF-κB, STAT3-associated, and inflammasome pathways, thereby contributing to several hallmarks of cancer [54,57,58]. Persistent inflammatory signaling may enhance tumor-cell proliferation, resistance to apoptosis, epithelial–mesenchymal transition (EMT), angiogenesis, and metastatic potential [57,58]. Chronic DAMP release may promote tumor progression and immune evasion [54].

Simultaneously, DAMPs influence the recruitment and polarization of immune cells within the TME, including macrophages, neutrophils, dendritic cells, and myeloid-derived suppressor cells (MDSCs), thereby modulating antitumor immunity [59,60].

DAMPs emitted during immunogenic cell death, particularly surface-exposed calreticulin, extracellular ATP, HMGB1, and annexin A1, can enhance antitumor immune responses by promoting the recruitment, activation, and antigen-presenting capacity of dendritic cells and subsequent cytotoxic T-cell responses [59].

From a translational perspective, DAMP-associated pathways are increasingly being investigated as potential biomarkers and therapeutic targets in oncology [61,62]. Circulating cfDNA, a DAMP-related analyte, has been investigated across malignancies as a potential marker of tumor burden, treatment response, and disease progression [63].

### 3.3. Evidence Regarding DAMPs in Thyroid Diseases

In thyroid cancer, increasing evidence suggests that DAMP-associated pathways may contribute to tumor development and progression. To facilitate interpretation of the heterogeneous evidence base, the mechanistic findings discussed below are distinguished as human thyroid-disease evidence, human thyroid-cancer evidence, thyroid-specific preclinical evidence, or evidence extrapolated from other malignancies and general immunology.

#### 3.3.1. HMGB1

HMGB1 is one of the best-characterized DAMPs implicated in sterile inflammation, autoimmunity, and cancer biology. Initially identified as a chromatin-associated nuclear protein involved in DNA organization and transcriptional regulation, HMGB1 is now recognized as an important extracellular alarmin released by stressed, damaged, or necrotic cells [64,65]. Its biological activity depends on its intracellular or extracellular localization and redox state. Extracellular HMGB1 may amplify inflammatory signaling through self-sustaining positive-feedback mechanisms, promoting further HMGB1 release from neighboring cells [66].

The best-characterized receptors mediating HMGB1-induced immune activation are TLR4 and RAGE [67]. Activation of these pathways promotes NF-κB signaling, cytokine production, leukocyte recruitment, and chronic inflammatory responses. HMGB1-related signaling has been implicated in experimental autoimmune thyroiditis [32], while increased HMGB1/RAGE expression has been demonstrated in orbital tissue from patients with Graves’ ophthalmopathy (GO) [68]. Human circulating data appear context-dependent: clinically stable levothyroxine-treated patients with HT showed lower serum HMGB1 than healthy controls [29], whereas circulating HMGB1 was higher in patients with GO than in patients with GD without ophthalmopathy [69].

Beyond autoimmunity, HMGB1 has attracted increasing interest in thyroid oncology. Experimental studies indicate that HMGB1 signaling may contribute to tumor progression, immune modulation, and resistance to targeted therapies [70].

HMGB1-associated signaling has been implicated in PTC and ATC biology, with experimental evidence linking it to proliferation, invasion, inflammatory remodeling, or resistance to targeted therapies [70,71,72].

Taken together, current evidence indicates that HMGB1-related signaling is involved in autoimmune thyroid inflammation, although circulating HMGB1 findings appear context-dependent across thyroid disorders. In thyroid cancer, HMGB1 may additionally contribute to tumor progression and resistance to targeted therapy. These observations support further evaluation of HMGB1 as a context-dependent candidate biomarker and therapeutic target; however, current thyroid-specific evidence does not establish independent prognostic value or clinically validated prognostic utility.

#### 3.3.2. S100 Proteins and Calprotectin

Calprotectin, a heterodimer composed of S100A8 and S100A9 proteins, also known as MRP8 and MRP14, respectively, represents another clinically relevant DAMP involved in inflammatory and neoplastic processes. Released predominantly by activated neutrophils and monocytes, calprotectin functions as a potent pro-inflammatory mediator and amplifier of innate immune signaling [73]. Through interactions with PRRs, particularly TLR4, S100A8/A9 promotes cytokine production, leukocyte recruitment, and sustained inflammatory activation [74,75].

Circulating S100A8/A9 concentrations are closely associated with neutrophil activation and systemic inflammatory burden. Elevated calprotectin levels have been reported in numerous acute and chronic inflammatory conditions and may also be influenced by cardiovascular and metabolic risk factors, including smoking, obesity, hyperglycemia, and dyslipidemia [76]. In thyroid diseases, increased serum calprotectin concentrations have been observed during the acute inflammatory phase of subacute thyroiditis, with levels declining during recovery [77]. These findings suggest that S100A8/A9 may reflect inflammatory activity and disease dynamics in thyroid inflammation.

Beyond inflammatory thyroid disorders, calprotectin has also emerged as a potential biomarker in thyroid oncology. Studies in PTC have demonstrated increased serum S100A8/A9 concentrations and associations with markers of oxidative stress [78]. S100A8/A9 signaling has been linked to oxidative stress in PTC, while its established effects on neutrophil activation and inflammatory amplification suggest a potential role in shaping the thyroid tumor microenvironment [74,78]. Emerging data additionally suggest that extracellular DNA and NET-associated components may function as secondary DAMPs, further amplifying inflammatory and protumorigenic signaling networks in aggressive thyroid malignancies [14,17,79]. Given the close relationship between oxidative stress, neutrophil activation, and tumor-associated inflammation, calprotectin warrants further investigation as a candidate marker linking innate immune activation with thyroid tumor biology.

#### 3.3.3. Serum Amyloid A

Serum amyloid A (SAA) proteins are acute-phase apolipoproteins synthesized predominantly by hepatocytes in response to inflammatory stimuli. Among the SAA family members, SAA1 and SAA2 are major components of the acute-phase response (APR), together with C-reactive protein (CRP) [80]. Their expression is strongly induced by pro-inflammatory cytokines, including interleukin-1 (IL-1), IL-6, and TNF-α.

Beyond their role as acute-phase reactants, SAA proteins may also function as DAMPs by activating innate immune signaling through TLR2, TLR4, and other PRRs [81]. Subsequent activation of inflammasome-associated pathways promotes the production of pro-inflammatory cytokines, including interleukin-1β (IL-1β) and interleukin-18 (IL-18), thereby amplifying local inflammatory responses.

Emerging evidence suggests a potential role for SAA in inflammatory thyroid disorders. Elevated serum SAA concentrations have been reported in patients with HT, supporting the hypothesis that SAA-associated signaling may contribute to persistent thyroid inflammation and autoimmune activation [82,83]. Although the underlying mechanisms in thyroid disease remain incompletely understood, SAA may contribute to inflammatory amplification through cytokine and chemokine induction, leukocyte recruitment, and modulation of innate immune responses [84].

Given its close association with systemic inflammation and innate immune activation, SAA represents a potentially relevant biomarker candidate in inflammatory thyroid diseases.

#### 3.3.4. Heat Shock Proteins

Heat shock proteins (HSPs) are stress-responsive molecular chaperones involved in protein folding, stabilization, and cellular adaptation to adverse conditions, including oxidative stress, hypoxia, infection, and inflammation [85]. In addition to their intracellular cytoprotective functions, extracellular HSPs may act as DAMPs capable of modulating innate immune responses and inflammatory signaling pathways [37].

Importantly, the thyroid-cancer-specific studies discussed below primarily evaluated intracellular HSP expression and chaperone-related functions. Studies of HSP27, TRAP1, and HSP90 in thyroid cancer did not directly assess extracellular HSP release or receptor-dependent DAMP signaling; therefore, their findings should not be interpreted as direct evidence of HSP-mediated DAMP activity in thyroid cancer [86,87,88].

Among HSP family members, increasing attention has been directed toward heat shock protein family A member 4 (HSPA4). Experimental and bioinformatic studies suggest that HSPA4 expression is upregulated in several malignancies, including thyroid carcinoma [89]. HSP-associated pathways may contribute to tumor progression through stabilization of oncogenic proteins, modulation of resistance to apoptosis, and interactions with inflammatory signaling networks [90].

Although evidence regarding HSPA4 in thyroid diseases is limited, emerging findings support further investigation of HSP-associated pathways as potential biomarkers and therapeutic targets in thyroid cancer.

#### 3.3.5. Decorin

Decorin (DCN) is a small extracellular matrix proteoglycan involved in tissue organization, collagen fibrillogenesis, and the regulation of cellular signaling pathways. In addition to its structural functions, DCN may act as a DAMP and participate in sterile inflammation and the modulation of innate immune responses [91].

Increasing evidence supports a tumor-suppressive role for DCN in several malignancies. Experimental studies have demonstrated that DCN may inhibit tumor-cell proliferation, angiogenesis, metastatic dissemination, and inflammatory remodeling of the TME [92]. In thyroid cancer, reduced DCN expression has been reported in FTC, suggesting that DCN may function as a tumor suppressor in this malignancy [93].

Mechanistically, DCN-associated pathways appear to influence extracellular matrix organization, oncogenic signaling, angiogenesis, and cellular senescence [94,95]. Preclinical studies further suggest that DCN may suppress PTC progression through modulation of the epidermal growth factor receptor (EGFR)/Src homology 2 domain-containing protein tyrosine phosphatase 2 (SHP2)/FER tyrosine kinase signaling axis and alterations in endosomal trafficking [93,96].

Although current evidence remains largely experimental, DCN-associated pathways represent an emerging area of interest in thyroid cancer biology and may offer future opportunities for biomarker development and targeted therapeutic strategies [96,97].

Whether DAMP-related biomarkers provide incremental value beyond established molecular diagnostics and clinicopathological risk stratification remains to be determined [98,99].

However, despite growing interest in DAMP-mediated oncogenic signaling, thyroid-specific evidence remains limited and largely experimental. Further prospective studies are required to determine the diagnostic specificity, prognostic value, and therapeutic relevance of DAMP-associated biomarkers in thyroid malignancies.

The pro-tumor effects of selected DAMP-related molecules in thyroid cancer are summarized in Table 1 and Table 2.

## 4. Neutrophil Extracellular Traps in Thyroid Diseases: Mechanistic and Translational Perspectives

### 4.1. What Are NETs and Why Do They Matter Clinically?

NETs are extracellular, web-like structures released by activated neutrophils and composed primarily of decondensed nuclear or mitochondrial DNA, histones, and granule-derived proteins, such as myeloperoxidase (MPO) and neutrophil elastase (NE). Histones incorporated into NETs are frequently citrullinated through the activity of peptidylarginine deiminase 4 (PAD4), which promotes chromatin decondensation and facilitates the extracellular release of DNA. Consequently, extracellular chromatin becomes decorated with citrullinated histones and neutrophil granule proteins, forming the characteristic NET architecture [107,108].

Initially described as an antimicrobial defense mechanism, NETs are now recognized as broader effectors of innate immunity with clinically relevant collateral consequences, including endothelial injury, thrombosis, autoantigen exposure, and tumor-promoting inflammation. From a biomarker perspective, NET biology can be assessed using circulating analytes with different degrees of specificity. Composite extracellular DNA–neutrophil protein complexes, such as MPO–DNA or NE–DNA, provide greater biological specificity for neutrophil-derived extracellular traps. Citrullinated histone H3 (CitH3) reflects PAD4-dependent nuclear NET formation but does not capture all NET phenotypes, particularly mitochondrial DNA-derived traps [16]. In contrast, total cfDNA and nucleosomes are nonspecific markers that may also originate from other forms of cellular injury or death.

### 4.2. NETosis Pathways: Classical, Vital, and Mitochondrial DNA Traps

The NET-release pathways summarized below are established primarily from general neutrophil biology. Their relative contribution to thyroid disease remains incompletely defined, with direct thyroid-specific mechanistic evidence currently strongest for mitochondrial extracellular DNA trap formation in ATC.

Mechanistically, NET release can proceed through at least three partially overlapping pathways.

1. Classical NETosis

Classical, often termed “suicidal,” NETosis typically involves NADPH oxidase 2 (NOX2)-dependent ROS generation, nuclear-envelope disintegration, chromatin decondensation, and eventual cell-membrane rupture. A key molecular step is PAD4-mediated histone citrullination, which facilitates chromatin relaxation and extracellular expulsion. PAD4, encoded by PADI4, is considered a central enzymatic regulator of NETosis. It catalyzes the conversion of histone arginine residues into citrulline, thereby disrupting electrostatic interactions between histones and DNA. This process promotes chromatin decondensation, a hallmark of NET formation [109]. Unlike apoptosis or necrosis, classical NETosis is characterized by extensive chromatin remodeling, loss of nuclear architecture, and extracellular chromatin release [110,111]. Among the histone modifications implicated in NET formation, citrullination remains the best characterized and forms the basis for the widespread use of CitH3 as a NET-associated biomarker [109,110].

2. Vital NET Release

Vital NET release allows neutrophils to remain viable and retain functional activity following vesicular extrusion of decondensed chromatin. Such non-lytic NET release may be particularly relevant in settings of persistent or repeated neutrophil activation, including chronic inflammatory conditions and potentially the tumor microenvironment [112].

3. Mitochondrial Extracellular DNA Traps

A distinct form of extracellular trap release involves the extrusion of mitochondrial DNA (mtDNA) by viable neutrophils without the extensive nuclear disintegration and cell death characteristic of classical NETosis. This mechanism may be particularly relevant in aggressive thyroid malignancies. In a recent study, soluble mediators released by ATC cells were shown to induce the formation of mtDNA-containing extracellular traps by neutrophils [79]. This process was dependent on ROS, including mitochondrial ROS, but occurred independently of neutrophil death. Moreover, neutrophils exposed to ATC-conditioned medium promoted ATC-cell proliferation, and this effect was reduced following NET degradation, suggesting that extracellular traps may actively support tumor growth within the ATC microenvironment [79].

### 4.3. Measuring NETs: Clinically Applicable Methods and Their Limitations

The above-mentioned findings also have implications for NET biomarker selection. CitH3 reflects PAD4-mediated histone citrullination and is therefore most informative for NET formation involving nuclear chromatin decondensation. It should not, however, be regarded as a universal marker of all extracellular trap phenotypes. Mitochondrial extracellular DNA traps may form without nuclear chromatin decondensation or histone citrullination and may therefore generate little or no CitH3 signal. This distinction may be particularly relevant to ATC, because ATC-derived soluble mediators have been shown experimentally to induce ROS-dependent, cell-death-independent release of mtDNA-containing extracellular traps [79]. Consequently, reliance on CitH3 alone could underestimate NET activity in aggressive thyroid cancers if mitochondrial trap formation represents a substantial component of the neutrophil response. The magnitude of this potential underestimation in patients with ATC is currently unknown, because the available thyroid-specific evidence is primarily experimental rather than derived from direct in vivo quantification of mitochondrial NETs. Future studies in aggressive thyroid cancers should therefore combine pathway-specific markers of nuclear NET formation with orthogonal approaches capable of detecting mitochondrial DNA release, such as mtDNA-specific molecular assays together with evidence of neutrophil origin and structural NET assessment [107].

For translational interpretation, circulating biomarkers related to NET biology should be distinguished according to their biological and analytical specificity. Relatively NET-specific or pathway-informed markers include composite extracellular DNA–neutrophil protein complexes, such as MPO–DNA and NE–DNA, as well as CitH3, which reflects PAD4-dependent nuclear chromatin decondensation. However, these markers also have important limitations: MPO–DNA assays may be affected by nonspecific and artifactual signals, whereas CitH3 does not capture all NET phenotypes, particularly mitochondrial extracellular DNA traps. NET-associated but nonspecific markers include total cfDNA/dsDNA, nucleosomes, extracellular histones, and isolated neutrophil granule proteins, because these analytes may also originate from apoptosis, necrosis, tissue injury, inflammation, or cellular turnover independently of NET formation. General DAMP and inflammatory markers, including HMGB1, S100A8/A9, SAA, and HSPs, primarily reflect cellular stress and innate immune activation rather than NETosis itself. Accordingly, no single circulating analyte should be interpreted as a direct quantitative measure of NET formation without appropriate biological context and orthogonal evidence [107,113,114].

cfDNA, particularly circulating double-stranded DNA (dsDNA), is one of the most frequently studied circulating analytes associated with NET biology; however, it is not specific for NET formation. Elevated circulating cfDNA concentrations have also been reported in thyroiditis and benign thyroid diseases, including multinodular goiter (MNG) [115]. Importantly, one clinical study found no significant differences in cfDNA concentrations between patients with thyroiditis, benign thyroid disease, and thyroid malignancy, further highlighting the limited disease specificity of total cfDNA [115]. Consequently, total cfDNA concentration should not currently be considered a disease-specific marker of thyroid malignancy or NET formation. Its elevation in malignant, benign, and inflammatory thyroid conditions limits its diagnostic utility when measured in isolation. Rather than advocating its immediate inclusion in a clinical multimarker panel, future studies should determine whether cfDNA provides incremental value when assessed under standardized pre-analytical conditions together with orthogonal molecular or NET-associated markers.

Analytical validity currently represents a major barrier to the clinical translation of circulating NET biomarkers. In particular, MPO–DNA ELISA measurements in human plasma are susceptible to non-specific antibody interactions and artifactual signals and may not reliably reflect NET formation in vivo [113]. Therefore, MPO–DNA complexes should currently be regarded as exploratory research measurements rather than validated clinical biomarkers. Pre-analytical factors, including anticoagulant selection, time to processing, centrifugation protocols, sample storage, and repeated freeze–thaw cycles, can further influence circulating DNA- and NET-associated measurements and substantially limit inter-study comparability [107,108,114]. Multimarker assessment cannot overcome these limitations unless sample handling and analytical procedures are first harmonized and prospectively validated.

In tumor tissue, NETs are commonly assessed by immunofluorescence or immunohistochemistry, using combinations of extracellular DNA and neutrophil-associated markers such as MPO, NE, and CitH3 to improve the specificity of NET identification [107,116]. Spatial tissue analysis may further characterize the localization of NETs relative to tumor cells, stromal compartments, vasculature, and invasive or thrombotic regions [117].

Tissue NET burden has been associated with clinically relevant tumor phenotypes and adverse oncological outcomes, while circulating NET-associated markers provide complementary systemic information [116]. NET-rich tumor microenvironments may also contribute to immunosuppression, invasion, metastasis, and therapeutic resistance [117]. Together, tissue and circulating NET measurements may therefore provide complementary information for the assessment of tumor aggressiveness and prognosis.

### 4.4. NETs in Autoimmune Thyroid Disease (AITD): What We Know and What’s Missing

Evidence regarding NET involvement in autoimmune thyroid diseases (AITDs) is limited and relates predominantly to HT. In a small clinical study, patients with HT exhibited increased circulating levels of NE and proteinase 3, together with an enhanced propensity of neutrophils to undergo NETosis. Proteinase 3 levels were also associated with thyroid autoantibody titers, suggesting a potential link between neutrophil activation and thyroid-specific autoimmunity [118].

Serum obtained from patients with HT enhanced NET formation in neutrophils from healthy donors, indicating that circulating disease-associated factors may promote a pro-NETotic phenotype [118]. NET-associated extracellular DNA and granule proteins may, in turn, increase autoantigen availability and reinforce interactions between innate and adaptive immune responses [118,119].

However, current evidence is derived mainly from small cohorts and experimental studies. The relationships between circulating NET-associated markers, local NET formation within thyroid tissue, disease activity, thyroid function, and treatment response remain poorly defined. Moreover, direct evidence in GD and GO is scarce. Larger prospective studies are required to determine whether NET-associated markers provide clinically relevant information beyond conventional thyroid autoantibodies and hormonal measurements.

### 4.5. NETs in Thyroid Cancer: Current Evidence and Biomarker Signals

NETs are increasingly recognized as functional components of the TME. In multiple solid tumors, NETs have been mechanistically associated with:

Pre-metastatic niche formation and the trapping of circulating tumor cells;
extracellular matrix remodeling mediated by proteases, including NE and matrix metalloproteinases (MMPs), as well as extracellular histones;
prothrombotic states and the risk of venous thromboembolism (VTE);
context-dependent immune modulation, including the suppression or diversion of effective T-cell responses.

Recent reviews have synthesized these mechanisms and highlighted associations between NET-related analytes and adverse clinicopathological features across several malignancies. However, such associations should not be interpreted as evidence of independent prognostic or predictive value unless supported by longitudinal and multivariable validation. However, NET-targeting strategies must account for pathway heterogeneity and the potential risk of impairing antimicrobial host defense [11,14].

Circulating cfDNA levels may reflect increased cellular turnover, inflammation, and extracellular chromatin release, but are not specific to NET formation [63]. In studies involving patients with DTC, including individuals previously treated with surgery and/or radioactive iodine (RAI), significantly increased circulating dsDNA levels were observed compared with healthy controls [14]. These findings suggest a potential role for cfDNA as a biomarker of persistent or recurrent disease activity in thyroid cancer.

Recent evidence suggests that elevated circulating CitH3 levels may correlate with the presence and progression of cancer, including thyroid malignancies [15]. Increased serum CitH3 concentrations likely reflect enhanced neutrophil activation and intensified inflammatory signaling within the TME [16,120]. Importantly, serum CitH3 levels were significantly higher in patients with DTC, including PTC and FTC, than in healthy controls and patients with MNG [14]. These findings support the potential utility of CitH3 as a candidate biomarker of thyroid cancer-associated NETosis.

More aggressive thyroid cancer phenotypes, particularly dedifferentiated thyroid carcinoma (De-DTC), exhibited higher levels of several circulating analytes associated with NET biology. These findings are consistent with increased neutrophil-related inflammatory activity, but the elevation of nonspecific markers such as dsDNA or nucleosomes should not, in isolation, be interpreted as direct evidence of increased NET formation [121,122].

A clinically oriented study evaluated a panel of circulating analytes related to NET biology in cohorts including DTC, De-DTC, MNG, and healthy controls. The panel comprised nonspecific markers such as dsDNA and nucleosomes, together with the more pathway-associated CitH3 and composite MPO–DNA complexes, as well as neutrophil-related mediators, including MPO, pentraxin 3 (PTX3), matrix metalloproteinase 9 (MMP-9), C-X-C motif chemokine ligand 8 (CXCL8), and granulocyte–macrophage colony-stimulating factor (GM-CSF) [14]. The principal translational observations were as follows:

Several circulating analytes associated with NET biology were increased, particularly in De-DTC compared with healthy controls; CitH3 showed a more discriminative pattern, with increased levels in both De-DTC and DTC compared with healthy controls and patients with MNG;

MPO–DNA complexes were selectively increased in De-DTC compared with DTC, MNG, and healthy controls, whereas nucleosome levels did not significantly distinguish De-DTC from DTC [14].

A key mechanistic study investigating NET formation in thyroid cancer revealed that soluble mediators released by ATC cell lines induced extracellular trap formation in neutrophils isolated from healthy donors when using conditioned media. In contrast, conditioned media derived from PTC cells or normal thyroid cells did not induce a comparable response [79].

ATC-induced extracellular trap release occurred in an ROS-dependent manner and was associated with increased mitochondrial ROS production. Importantly, the neutrophils remained viable, and the released extracellular traps contained predominantly mtDNA, indicating a cell death-independent mechanism distinct from classical lytic NETosis. Fluorescence microscopy, flow cytometry, and molecular analyses confirmed the mitochondrial origin of the extracellular DNA [79].

The study also provided functional evidence that these structures may contribute to tumor progression. Neutrophils exposed to ATC-conditioned medium enhanced ATC-cell proliferation, whereas degradation of extracellular traps attenuated this effect. These findings suggest a bidirectional interaction in which ATC-derived mediators activate neutrophils, while neutrophil-derived extracellular traps subsequently support tumor-cell growth [79].

NETs may therefore represent active components of the inflammatory TME rather than merely secondary markers of neutrophil activation [117]. However, these findings were obtained predominantly from cell-line-based in vitro models and require confirmation in primary human tumors and clinical cohorts [79].

Neutrophils are increasingly recognized as a heterogeneous and functionally plastic cell population rather than a uniform component of the TME. In thyroid cancer, their phenotype may vary according to maturation stage, density, activation status, transcriptional profile, and exposure to tumor-derived mediators. Patients with undifferentiated thyroid cancer exhibited a higher proportion of circulating immature high-density neutrophils and a lower proportion of mature high-density neutrophils than patients with differentiated thyroid cancer. In addition, programmed death-ligand 1 (PD-L1) expressing immature neutrophils were enriched in patients with advanced disease, suggesting that selected neutrophil subsets may contribute to immunosuppressive mechanisms and impaired antitumor immunity. Transcriptomic analyses further indicated that neutrophil alterations were associated with the downregulation of pathways related to innate immune responses and T-cell receptor signaling. These findings support the concept that neutrophil heterogeneity may reflect clinically relevant differences in tumor biology and may be associated with reduced T-cell-mediated immune activity in more aggressive thyroid cancer phenotypes [123].

The functional properties of neutrophils may also be directly shaped by the thyroid TME. Soluble factors released by thyroid cancer cells enhanced neutrophil chemotaxis and survival and induced an activated phenotype characterized by increased expression of CD11b and CD66b, CD62L shedding, ROS production, and the release of pro-inflammatory, angiogenic, and matrix-remodeling mediators, including CXCL8, vascular endothelial growth factor A (VEGF-A), TNF-α, and MMP-9. Moreover, increased tumor-associated neutrophil infiltration was correlated with larger tumor size, supporting a potential association between neutrophil activation and tumor progression [13].

Within this broader framework, NET formation should be regarded as one of several functional responses adopted by tumor-associated neutrophils. Depending on their maturation and activation states, neutrophils may promote inflammation, angiogenesis, extracellular matrix remodeling, immune suppression, or extracellular trap formation. Characterization of these distinct neutrophil states may therefore provide clinically relevant information regarding tumor aggressiveness, immune dysregulation, and disease progression. DAMP- and NET-related mechanisms may exert opposing and context-dependent effects on thyroid cancer biology. To distinguish established thyroid-specific findings from broader mechanistic hypotheses, their predominant pro- or anti-tumor functions and the corresponding levels of evidence are summarized in Table 3.

## 5. Diagnostic and Prognostic Potential of NETs and DAMPs in Thyroid Diseases

The growing recognition of innate immune activation in thyroid diseases has generated increasing interest in DAMP- and NET-associated molecules as candidate biomarkers with potential diagnostic and prognostic relevance. Unlike conventional thyroid diagnostics, which primarily evaluate hormonal function, autoantibody status, imaging characteristics, and cytological morphology, DAMP- and NET-related markers may provide insight into inflammatory activity, TME remodeling, and disease aggressiveness. Importantly, several of these molecules can be detected in peripheral blood, making them attractive candidates for minimally invasive liquid biopsy approaches. Importantly, biological or clinicopathological associations should not be equated with demonstrated diagnostic performance; for most DAMP- and NET-associated biomarkers, validated cutoffs, sensitivity, specificity, predictive values, external validation, and incremental diagnostic value over established thyroid diagnostics remain unavailable.

Similarly, different levels of prognostic evidence should be distinguished. An association with disease phenotype refers to cross-sectional relationships with features such as dedifferentiation, metastatic presentation, or disease severity. A prognostic association requires a longitudinal relationship between a biomarker and a subsequent clinical outcome. Independent prognostic value additionally requires demonstration of this association after adjustment for established prognostic factors in an appropriate multivariable model. Clinically validated prognostic utility further requires reproducibility in independent cohorts and evidence that incorporation of the biomarker meaningfully improves prognostic assessment or clinical decision-making.

To distinguish biological and mechanistic associations from biomarkers that have undergone direct clinical evaluation, the principal human studies assessing DAMP- and NET-related analytes in thyroid diseases are summarized in Table 4, including study populations, analytical methods, diagnostic-performance parameters, validation status, and major limitations.

HMGB1 has emerged as a translational biomarker candidate in thyroid diseases, although circulating findings are heterogeneous across clinical contexts. In clinically stable levothyroxine-treated HT, serum HMGB1 was lower than in healthy controls [29], whereas higher circulating HMGB1 levels were reported in patients with GO compared with GD without ophthalmopathy [69]. In thyroid cancer, increased tissue HMGB1 expression has been associated with more aggressive clinicopathological features and malignant progression [101,102,128].

S100A8/A9, or calprotectin, is another DAMP-associated molecule with potential clinical relevance in thyroid pathology. Because S100A8/A9 is closely linked to neutrophil activation and inflammatory signaling, elevated circulating concentrations may reflect systemic inflammatory burden and tumor-associated innate immune activation. Preliminary evidence suggests associations between S100A8/A9 levels, oxidative stress pathways, and PTC biology. Furthermore, S100 proteins are increasingly recognized as mediators of cancer-associated inflammation, angiogenesis, and immune modulation, raising the possibility that they may contribute to aggressive tumor phenotypes and metastatic progression. Nevertheless, thyroid-specific clinical evidence remains relatively limited, and additional prospective studies are required to validate their diagnostic and prognostic utility [103,104,106].

Circulating analytes investigated in relation to NET biology differ substantially in specificity. Total cfDNA/dsDNA and nucleosomes are nonspecific measures of extracellular DNA or chromatin and, when measured in isolation, do not establish NET formation. In contrast, composite DNA–neutrophil protein complexes, such as MPO–DNA or NE–DNA, and CitH3 provide more pathway-informed evidence of NET biology, although these measurements also have important analytical limitations.

Circulating cfDNA measurements should be interpreted according to their analytical target. Total cfDNA concentration is a nonspecific measure of extracellular DNA derived from multiple cellular sources and does not identify either tumor-derived DNA or NET formation [115,129]. In contrast, tumor-informed cfDNA assays based on cancer-associated mutation or methylation patterns assess tumor-derived molecular signals rather than NETosis [127,130]. The ThyMet classifier, for example, uses a defined cfDNA methylation signature to discriminate PTC from benign thyroid nodules [127]; its diagnostic performance should therefore not be interpreted as evidence of NET formation. Total cfDNA has also been evaluated in the postoperative assessment of disease status in DTC. In a prospective single-center study of 254 patients, plasma cfDNA levels were only slightly higher in patients with active structural disease than in disease-free patients, and the difference was not statistically significant (*p* = 0.122) [122]. These findings do not establish a longitudinal prognostic association or independent prognostic value and highlight the limited utility of total cfDNA as a standalone prognostic biomarker. However, substantial overlap between malignant, benign, and inflammatory thyroid conditions remains a major limitation of total cfDNA concentration as a diagnostic measure. Elevated total cfDNA may also occur in non-malignant inflammatory and systemic conditions, thereby limiting its disease specificity [115,129].

A potential future application of DAMP- and NET-related biomarkers is their integration into the diagnostic assessment of indeterminate Bethesda III/IV thyroid nodules. However, no DAMP- or NET-based biomarker panel has been clinically validated for this indication, and its incremental value beyond ultrasound risk stratification, cytology, and established molecular testing remains unknown [18,25]. Their use should therefore be regarded as hypothesis-generating and would require prospective thyroid-specific validation before any clinical implementation could be considered.

A particularly important limitation in this setting is the coexistence of autoimmune thyroiditis. DAMP-associated biomarkers such as HMGB1, S100A8/A9, and total cfDNA reflect cellular stress, tissue injury, and inflammatory activation and are therefore not inherently malignancy-specific. At present, no validated concentration cutoff or composite specificity threshold has been established for these biomarkers to distinguish PTC from benign autoimmune thyroiditis, particularly in patients with coexisting HT and Bethesda III/IV cytology. Accordingly, these markers should not currently be used to discriminate benign autoimmune inflammation from malignancy or to guide surgical decision-making. Future prospective studies should specifically stratify patients according to HT status and evaluate Bethesda III/IV nodules against histopathological reference standards using standardized assays, predefined diagnostic thresholds, and independent validation cohorts. Importantly, any proposed DAMP-based panel should demonstrate incremental diagnostic value beyond ultrasound risk stratification, cytology, and established molecular testing [25,26,29,78,115,129].

DAMP- and NET-related biomarkers have been associated with adverse clinicopathological phenotypes in thyroid malignancies, but current evidence does not establish their prognostic value. In patients with thyroid cancer, circulating analytes investigated in relation to NET biology, including nonspecific dsDNA and nucleosomes, pathway-associated CitH3, and composite MPO–DNA complexes, have been reported to be elevated particularly in dedifferentiated disease, with several analytes showing cross-sectional associations with metastatic presentation and greater disease severity [14]. These findings should be interpreted as associations with disease phenotype rather than as evidence that these biomarkers predict subsequent progression, recurrence, survival, or other clinical outcomes. More broadly, NETs have been implicated in tumor invasion, metastasis, immune evasion, treatment resistance, and cancer-associated thrombosis across cancer biology [15]; however, these mechanistic associations do not establish prognostic performance in thyroid cancer. At present, thyroid-specific evidence demonstrating longitudinal prognostic associations, independent prognostic value after multivariable adjustment, or clinically validated prognostic utility remains lacking. Similarly, whether NET-related analytes can predict future thrombotic events independently of established clinical risk factors in thyroid cancer remains to be determined.

From a therapeutic-monitoring perspective, tumor-informed cfDNA assays based on cancer-associated mutation or methylation signals are being investigated as indicators of tumor burden, progression, and treatment response in thyroid cancer [130]. These assays reflect tumor-derived molecular information and should not be interpreted as measures of NET formation.

In contrast, serial assessment of DAMP- and NET-related analytes, including nonspecific total cfDNA/nucleosomes and pathway-informed markers such as MPO–DNA complexes, remains investigational. Current thyroid-specific evidence largely reflects associations with disease phenotype rather than established prognostic associations. Prospective longitudinal studies with standardized assays, prespecified clinical outcomes, and multivariable adjustment for established prognostic factors are required to establish prognostic associations and independent prognostic value, followed by external validation before clinically validated prognostic utility can be claimed [107,113,114].

### Limitations of NET- and DAMP-Related Biomarkers

Despite their considerable translational potential, several important limitations currently restrict the clinical implementation of DAMP- and NET-related biomarkers. First, several candidate biomarkers, including cfDNA, HMGB1, S100A8/A9, and nucleosomes, lack disease specificity and may be elevated in numerous inflammatory, autoimmune, cardiovascular, infectious, or neoplastic conditions [107,115,129]. Second, substantial methodological heterogeneity exists regarding sample collection, processing, assay standardization, and interpretation of results [107,114]. NET detection methods, particularly MPO–DNA ELISA-based approaches, are especially vulnerable to analytical variability, limited specificity in plasma, and potentially false-positive or artifactual signals [107,113]. Finally, thyroid-specific studies evaluating DAMP- and NET-related biomarkers remain relatively limited and are generally based on small or exploratory cohorts, which restricts generalizability and clinical translation [107].

Consequently, the future clinical utility of DAMP- and NET-associated biomarkers in thyroid diseases will likely depend on standardized multimarker panels integrated with imaging, cytology, molecular diagnostics, and clinicopathological risk stratification rather than on isolated biomarker measurements alone [107,114].

An additional limitation derives from the narrative design of the present review. Although a structured literature search was performed, study selection was not based on a formal systematic-review protocol, and no formal risk-of-bias assessment was conducted. Consequently, selection and interpretation bias cannot be excluded, particularly given the heterogeneity and limited availability of thyroid-specific studies.

## 6. Clinical Perspectives and Future Directions

Despite the growing body of evidence linking DAMPs and NETs with thyroid autoimmunity and thyroid cancer biology, their clinical implementation remains at an early stage. Nevertheless, available data suggest that innate immune-related biomarkers may eventually complement current diagnostic and prognostic strategies in thyroid diseases, particularly in areas where substantial clinical uncertainty persists [129,131].

One of the most promising future applications involves the integration of DAMP- and NET-associated biomarkers into the diagnostic workup of indeterminate thyroid nodules. Current clinical management of Bethesda III and IV lesions remains challenging despite advances in ultrasound-based risk stratification and molecular testing. Because chronic sterile inflammation and innate immune activation appear to contribute to thyroid tumorigenesis and TME remodeling, circulating inflammatory biomarkers such as HMGB1, CitH3, S100A8/A9, and cfDNA may provide additional biological information beyond cytomorphology alone. However, such multimarker strategies remain hypothesis-generating and should not yet be considered clinically applicable, particularly given the current lack of standardized pre-analytical procedures and analytically validated assays for several NET-associated biomarkers. Their potential incremental value in the diagnostic assessment of indeterminate thyroid nodules will require prospective evaluation using harmonized sample-processing protocols and independent thyroid-specific validation cohorts [107,114,129,131,132].

For prospective clinical validation, biomarker prioritization should reflect both biological specificity and the maturity of the available clinical evidence. Tumor-informed cfDNA assays, particularly methylation-based approaches such as ThyMet, currently provide the most developed diagnostic-performance data and should be evaluated separately from nonspecific total cfDNA measurements. Among NET-associated analytes, composite MPO–DNA complexes and CitH3 may be prioritized as pathway-informed markers, whereas total cfDNA, nucleosomes, HMGB1, and S100A8/A9 should be considered exploratory adjuncts because of their limited disease specificity. Plasma should be used for cfDNA-based molecular assays, while a single prespecified biological matrix should be used consistently for protein- and NET-associated measurements. Pre-analytical procedures should be harmonized with respect to collection tubes, time to processing, centrifugation, storage conditions, and freeze–thaw cycles. Diagnostic thresholds should be prespecified or derived in a training cohort and locked before independent validation rather than transferred directly from small exploratory studies.

Such evaluation should preferably involve a prospective, multicenter diagnostic-accuracy study enrolling consecutive patients with Bethesda III/IV nodules. Biomarker assays and diagnostic thresholds should be prespecified, and an extended multimarker model incorporating prespecified DAMP- and NET-related analytes should be compared with a baseline model including clinical variables, ultrasound risk stratification, Bethesda cytology, and currently available molecular classifiers, with the primary objective of determining incremental diagnostic value. Diagnostic performance should be assessed against definitive histopathology or appropriately defined longitudinal follow-up in nonsurgical cases and should include measures of discrimination, calibration, sensitivity, specificity, predictive values, and clinical net benefit, followed by independent external validation. Only after demonstrating such incremental diagnostic value should clinical-impact studies assess whether biomarker-guided management can safely reduce diagnostic surgery without increasing the risk of missed malignancy.

Another clinically relevant direction concerns the identification of aggressive thyroid cancer phenotypes. Poorly differentiated thyroid carcinoma (PDTC), De-DTC, and ATC are characterized by highly inflammatory tumor microenvironments, increased neutrophil infiltration, oxidative stress, and immune dysregulation. DAMP- and NET-associated biomarkers may therefore help identify patients with biologically aggressive disease, increased metastatic potential, or resistance to targeted therapies. In this context, standardized liquid-biopsy approaches combining cfDNA analysis with molecular or inflammatory biomarkers warrant prospective investigation for disease monitoring and risk stratification; however, their diagnostic utility has not yet been established [122,129].

The relationship between DAMPs, NETs, and immunothrombosis also represents an important translational area. NET-associated components, including extracellular DNA and CitH3, contribute to endothelial injury, platelet activation, and thrombus formation. Consequently, future studies should evaluate whether NET-associated biomarkers could improve thrombosis risk stratification in patients with thyroid cancer, particularly in advanced disease characterized by extensive inflammatory activation [11,133].

Given the limited disease specificity of many DAMP- and NET-associated analytes, future models should evaluate their incremental value alongside imaging, cytology, molecular profiling, and clinicopathological variables rather than as isolated biomarkers [56,103,134].

From a therapeutic perspective, increasing attention is being directed toward targeting DAMP- and NET-mediated signaling pathways. However, evidence supporting these strategies in thyroid diseases currently derives predominantly from thyroid-specific preclinical studies or is extrapolated from broader cancer and immunology research, while direct clinical evidence in thyroid cancer remains lacking. Thyroid-specific preclinical evidence is available for NET degradation: in an ATC–neutrophil experimental model, degradation of extracellular traps attenuated NET-dependent stimulation of tumor-cell proliferation [79]. DNase-based degradation of established NETs therefore represents a potential therapeutic approach, although its antitumor use in thyroid cancer has not been clinically evaluated and broader evidence is largely extrapolated from other malignancies [15]. In contrast, PAD4 inhibition is supported primarily by general NET biology and preclinical studies outside thyroid cancer; although PAD4 inhibitors can suppress NET formation, direct thyroid-cancer-specific therapeutic evidence is currently lacking [126]. Similarly, antagonism of HMGB1-associated TLR and RAGE signaling is mechanistically plausible, but therapeutic evidence in thyroid cancer remains limited and is largely extrapolated from broader DAMP, cancer, and immunology research [56,62,100]. Importantly, NETs and pattern-recognition receptor pathways also contribute to physiological antimicrobial host defense. Consequently, systemic or prolonged inhibition of NET formation or DAMP-sensing pathways may impair protective innate immune responses and potentially increase susceptibility to infection, particularly in immunocompromised patients [11,15]. These approaches should therefore be regarded as investigational and require thyroid-specific in vivo validation, careful assessment of infectious risk, and subsequent clinical evaluation.

## 7. Conclusions

Finally, additional prospective longitudinal studies involving larger patient cohorts are required to determine the clinical utility of DAMP- and NET-associated biomarkers in thyroid diseases. Future investigations should evaluate their diagnostic sensitivity and specificity, prognostic value, relationship with treatment response, and potential integration into existing thyroid cancer management algorithms.

Taken together, DAMPs and NETs represent a rapidly evolving field at the intersection of inflammation, autoimmunity, and thyroid oncology. However, current evidence remains limited by low disease specificity, methodological heterogeneity, small exploratory cohorts, and the lack of independent clinical validation. DAMP- and NET-associated biomarkers should therefore be regarded as investigational and should not currently influence routine diagnostic, prognostic, or therapeutic decision-making outside research settings. Further prospective, standardized, and externally validated studies are required before their integration into clinical practice can be considered.

## Figures and Tables

**Table 1:** DAMP-related mechanisms in thyroid cancer: initiation and progression.

Molecule	Initiation	Progression
HMGB1	Chronic extracellular HMGB1 signaling through PRRs, particularly TLR4 and RAGE, can sustain NF-κB-dependent inflammation and generate a tumor-promoting microenvironment [54,56,64,65,66,67,68,100]. Direct evidence that HMGB1 initiates thyroid cancer remains insufficient. Evidence basis: EX—general DAMP/immunology evidence; no direct thyroid-cancer initiation evidence.	Supported in thyroid cancer: increased HMGB1 expression and HMGB1-associated signaling have been linked to PTC malignant progression, proliferation and invasive behavior [70,71,72,101,102]. Evidence basis: HTC + TSP—human PTC tissue/clinicopathological evidence supported by thyroid-specific experimental studies.
S100 proteins/calprotectin (S100A8/A9)	S100A8/A9 amplify innate inflammatory signaling through TLR4/RAGE and can create a tumor-promoting inflammatory environment [73,74,75,76,103,104]. In thyroid cancer, increased S100A8/A9/calprotectin has been demonstrated, but a causal role in tumor initiation has not been established [78,105]. Evidence basis: HTC + EX—human thyroid-cancer association supported by broader inflammatory/cancer biology; no direct evidence of tumor initiation.	Supported in thyroid cancer: S100A8–RAGE signaling activates p38, ERK1/2 and JNK, promotes ATC-cell proliferation and enhances tumor growth [105]. Increased circulating calprotectin has also been reported in PTC [78]. Evidence basis: HTC + TSP—human thyroid-cancer evidence together with thyroid-specific in vitro and in vivo evidence.
Heat shock proteins (HSPs)	HSPs have distinct intracellular and extracellular functions: intracellular HSPs act primarily as molecular chaperones [85], whereas extracellular HSPs may function as DAMPs [37]. However, the thyroid-cancer studies cited here evaluated intracellular HSP biology and did not demonstrate extracellular HSP release or receptor-dependent DAMP signaling [86,87,88]. Direct evidence for HSP-mediated DAMP signaling in thyroid-cancer initiation is therefore lacking. Evidence basis: TSP + EX—thyroid-specific intracellular HSP evidence with extracellular DAMP functions extrapolated from broader literature.	Thyroid-specific evidence concerns intracellular HSP functions: HSP27 promotes PTC-cell proliferation and resistance to apoptosis [86], while TRAP1 supports cell-cycle progression and survival in thyroid carcinoma cells [87]. Neither study evaluated extracellular HSP-mediated DAMP signaling. Evidence basis: TSP (in vitro) + EX—thyroid-specific cell-model evidence supported by broader HSP biology.

**Evidence categories:** HTC, human thyroid-cancer evidence; TSP, thyroid-specific preclinical evidence; EX, evidence extrapolated from other malignancies or general immunology. Multiple categories are indicated where applicable. **Abbreviations:** ATC, anaplastic thyroid carcinoma; ERK, extracellular signal-regulated kinase; HMGB1, high-mobility group box 1; HSP, heat shock protein; JNK, c-Jun N-terminal kinase; NF-κB, nuclear factor kappa B; PRR, pattern-recognition receptor; PTC, papillary thyroid carcinoma; RAGE, receptor for advanced glycation end products; TLR, Toll-like receptor; TRAP1, tumor necrosis factor receptor-associated protein 1.

**Table 2:** DAMP-related mechanisms in thyroid cancer: metastatic dissemination, immune escape, and therapy resistance.

Molecule	Metastatic Dissemination	Resistance to Immune Defence	Resistance to Therapies
HMGB1	Supported mainly at the invasion/progression level: HMGB1-associated pathways promote migration and invasion of thyroid cancer cells, but direct evidence for HMGB1-driven distant metastatic dissemination remains limited [71,72,102]. Evidence basis: TSP—predominantly thyroid-cancer cell-model evidence; direct human metastatic evidence remains insufficient.	HMGB1 can modulate inflammatory and immune responses through TLR/RAGE signaling and contribute to a tumor-supportive microenvironment [54,64,65,66,67,68,100]. Direct thyroid-cancer-specific evidence for HMGB1-mediated immune escape remains limited. Evidence basis: EX—primarily general immunology/cancer evidence.	Supported in thyroid cancer: HMGB1-mediated excessive autophagy contributes to vemurafenib resistance in BRAF V600E thyroid cancer cells [70]. Evidence basis: TSP—thyroid-specific in vitro evidence.
S100 proteins/calprotectin (S100A8/A9)	Supported in ATC: S100A8 knockdown reduces lung metastatic burden in an orthotopic ATC model [105]. Broader S100 biology also supports roles in inflammatory and metastatic signaling [103,104,106]. Evidence basis: TSP (in vivo) + EX—direct thyroid-specific animal-model evidence supported by broader cancer biology.	S100A8/A9 regulate neutrophil and myeloid-cell recruitment and inflammatory signaling through PRRs [73,74,75,76,103,104]. However, direct evidence that S100A8/A9 causes immune escape in thyroid cancer remains insufficient. Evidence basis: EX—general immunology/inflammation evidence; direct thyroid-cancer immune-evasion evidence is lacking.	S100A8 represents a potential therapeutic target in ATC because its suppression reduces tumor-promoting activity [105]. However, direct evidence that S100A8/A9 mediates resistance to a defined anticancer drug in thyroid cancer remains insufficient. Evidence basis: TSP—thyroid-specific therapeutic-targeting evidence, but no direct treatment-resistance evidence.
Heat shock proteins (HSPs)	Pharmacological inhibition of intracellular HSP90 chaperone function suppresses migration and invasion of thyroid cancer stem-like cells [88]. This study did not evaluate extracellular HSP release or DAMP-receptor signaling, and direct evidence for HSP-driven distant metastatic dissemination remains lacking. Evidence basis: TSP (in vitro)—migration/invasion evidence; no direct in vivo or human metastatic evidence.	Extracellular HSPs may modulate innate and adaptive immune signaling as DAMPs in broader biological settings [37]; however, thyroid-cancer-specific studies [86,87,88] did not assess extracellular HSP release or receptor-dependent immune signaling. Direct evidence for HSP-mediated immune escape in thyroid cancer is therefore lacking. Evidence basis: EX—general DAMP/immunology evidence.	Thyroid-specific evidence reflects intracellular chaperone functions: TRAP1 silencing increases sensitivity to paclitaxel-induced apoptosis [87], while HSP90 inhibition suppresses malignant functions of thyroid cancer stem-like cells [88]. These findings do not demonstrate extracellular HSP-mediated DAMP signaling. Evidence basis: TSP (in vitro)—thyroid-specific experimental evidence.

**Evidence categories:** TSP, thyroid-specific preclinical evidence; EX, evidence extrapolated from other malignancies or general immunology. Multiple categories are indicated where applicable. **Abbreviations:** ATC, anaplastic thyroid carcinoma; HMGB1, high-mobility group box 1; HSP, heat shock protein; RAGE, receptor for advanced glycation end products; TLR, Toll-like receptor.

**Table 3:** Context-dependent pro- and anti-tumor functions of DAMP- and NET-related mechanisms in thyroid cancer and the corresponding level of evidence.

Mechanism	Predominant Effect	Main Tumor/Immune Consequence	Thyroid-Specific Evidence	Evidence Basis/Level	References
HMGB1 signaling	Pro-tumor/context-dependent	Promotes inflammatory signaling, proliferation, invasion and treatment resistance; immune effects depend on receptor context and redox state	HMGB1-associated signaling demonstrated in thyroid cancer models; associations with PTC progression have also been reported	HTC + TSP—human thyroid-cancer tissue/clinicopathological associations + thyroid-specific in vitro evidence	[70,71,72,101,102]
S100A8/A9–RAGE signaling	Pro-tumor	Enhances ATC-cell proliferation, tumor growth and metastatic dissemination	S100A8/A9 overexpression demonstrated in human ATC; S100A8 suppression reduced tumor growth and lung metastasis	HTC + TSP—human thyroid-cancer tissue/circulating evidence + thyroid-specific in vitro and in vivo evidence	[78,105]
NET/mitochondrial extracellular trap formation	Pro-tumor	Neutrophil-derived extracellular traps enhance ATC-cell proliferation and tumor-supportive inflammatory signaling	ATC-conditioned medium induces ROS-dependent mtDNA trap release from human neutrophils	TSP—thyroid-specific in vitro evidence	[79]
NET-associated biomarkers/NET burden	Pro-tumor association	Higher NET-associated biomarker levels are associated with dedifferentiated and metastatic thyroid cancer	Increased CitH3, MPO–DNA and nucleosomes reported particularly in De-DTC and more severe disease	HTC—clinical cohort	[14]
Tumor-associated neutrophil activation/immunosuppressive phenotype	Pro-tumor	Promotes inflammatory, angiogenic and immunosuppressive TME; immature PD-L1+ neutrophils are enriched in aggressive disease	Altered neutrophil activation and phenotype demonstrated in thyroid cancer	HTC—human thyroid-cancer tissue/circulating and transcriptomic evidence	[13,123]
Immunogenic DAMP release (CRT, ATP, HMGB1, annexin A1)	Anti-tumor	Promotes dendritic-cell recognition, phagocytosis and subsequent antitumor immune responses	SHP2 inhibition in ATC induces an immunogenic phenotype and enhances antitumor immunity	TSP + EX—thyroid-specific in vitro/in vivo evidence supported by general cancer immunology	[55,59,124]
Decorin-associated signaling	Anti-tumor	Suppresses thyroid-cancer proliferation and oncogenic EGFR/SHP2/FER signaling	Reduced DCN in human PTC; DCN overexpression suppresses tumor growth in experimental models	HTC + TSP—human thyroid-cancer tissue evidence + thyroid-specific in vitro/in vivo evidence	[93,96,97]
NET degradation	Anti-tumor intervention	Removes tumor-supportive extracellular traps and attenuates neutrophil-mediated stimulation of tumor cells	NET degradation reduces ATC-cell proliferation promoted by activated neutrophils	TSP—thyroid-specific in vitro evidence	[79]
PAD4/NETosis inhibition	Putative anti-tumor	May reduce NET-dependent inflammation and immune suppression	Mechanistically supported in general cancer/NET biology, but direct thyroid-cancer evidence is currently lacking	EX—evidence from general NET biology and other malignancies; no direct thyroid-cancer evidence	[107,109,110,111,125,126]

**Abbreviations:** ATC, anaplastic thyroid carcinoma; ATP, adenosine triphosphate; CitH3, citrullinated histone H3; CRT, calreticulin; DAMP, damage-associated molecular pattern; DCN, decorin; EGFR, epidermal growth factor receptor; FER, FER tyrosine kinase; HMGB1, high-mobility group box 1; MPO–DNA, myeloperoxidase–DNA complexes; mtDNA, mitochondrial DNA; NET, neutrophil extracellular trap; PAD4, peptidylarginine deiminase 4; PD-L1, programmed death-ligand 1; RAGE, receptor for advanced glycation end products; SHP2, Src homology 2 domain-containing protein tyrosine phosphatase 2; TME, tumor microenvironment. **Evidence categories:** HTC, human thyroid-cancer evidence; TSP, thyroid-specific preclinical evidence; EX, evidence extrapolated from other malignancies or general immunology. Multiple categories are indicated where applicable.

**Table 4:** Human clinical studies of DAMP- and NET-related analytes and selected cfDNA assays in thyroid diseases.

Biomarker/Analyte	Thyroid Disease/Cancer Subtype	Patients and Controls	Biological Sample	Analytical Method	Primary Clinical Endpoint	Principal Finding/Effect Estimate	Diagnostic Performance	Independent Validation	Major Limitations	Ref.
HMGB1	Hashimoto’s thyroiditis	45 HT; 45 healthy controls	Serum	ELISA	Discrimination of HT from healthy controls	HMGB1 was significantly lower in clinically stable, levothyroxine-treated HT than in healthy controls (*p* < 0.001). Effect estimate: NR	AUC 0.802; sensitivity 75.6%; specificity 75.6%; cutoff 514 pg/mL	No	Cross-sectional; treated, clinically stable HT population; no external validation.	[29]
HMGB1	Graves’ disease with vs. without Graves’ ophthalmopathy	22 GO; 22 GD without GO	Plasma	ELISA (HMGB1 Express ELISA Kit)	Association with GO	Circulating HMGB1 was higher in patients with GO than in patients with GD without ophthalmopathy (median 15.5 vs. 2.3 pg/mL; *p* < 0.001). HMGB1 > 8.86 pg/mL was associated with prevalent GO: adjusted OR 7.06 (95% CI 1.61–36.1; *p* = 0.012)	Cutoff 8.86 pg/mL; sensitivity 81%; specificity 73%; AUC: NR	No	Small cross-sectional cohort; association rather than prospective prediction; no external validation.	[69]
S100A8/A9 (calprotectin)	Subacute thyroiditis	36 patients; paired acute and recovery measurements	Serum	Micro-ELISA using commercial kit; 450 nm	Disease activity and follow-up	Calprotectin decreased from 96.92 ng/mL during the acute inflammatory phase to 37.98 ng/mL during recovery (*p* < 0.001). For permanent hypothyroidism, OR 1.003 (95% CI 0.994–1.011; *p* = 0.544)	NR	No	Small cohort; no separate healthy control group; paired inflammatory-disease assessment rather than malignancy discrimination.	[77]
S100A8/A9 (calprotectin)	PTC	30 PTC; 30 healthy controls; PTC reassessed 1 month after thyroidectomy	Serum	Serum calprotectin assay	Association with PTC and postoperative change	Preoperative calprotectin was higher in PTC than in healthy controls and decreased significantly after thyroidectomy (*p* < 0.001 for both comparisons). Effect estimate: NR	NR	No	Small cohort; healthy rather than benign/thyroiditis controls; no external validation.	[78]
Serum amyloid A (SAA)	Euthyroid Hashimoto’s thyroiditis	30 HT; 30 healthy controls	Serum	Serum SAA measurement	Systemic inflammatory activity	Mean SAA was significantly higher in euthyroid HT than in healthy controls (*p* = 0.002). Effect estimate: NR	NR	No	Small cross-sectional preliminary study; non-specific acute-phase marker; no malignancy comparison.	[82]
dsDNA, nucleosomes, CitH3, MPO-DNA	DTC and dedifferentiated DTC	20 DTC; 26 De-DTC; 26 MNG; 22 healthy controls	Serum	PicoGreen dsDNA assay; nucleosome ELISA; CitH3 ELISA; MPO-DNA capture assay	Association with malignancy, dedifferentiation and metastatic status	All four analytes were increased in De-DTC vs. healthy controls; CitH3 was increased in DTC/De-DTC vs. MNG/controls; MPO-DNA was selectively increased in De-DTC vs. DTC, MNG and controls. Effect estimate: NR	NR	No	Small exploratory cohorts; no external validation; analytical limitations of circulating NET assays.	[14]
Total cfDNA	Thyroiditis, benign thyroid disease and thyroid malignancy	33 thyroiditis; 37 benign; 30 malignant; 21 healthy controls	Plasma	Quant-iT high-sensitivity DNA assay/Qubit fluorometry	Differentiation of thyroid disease groups	cfDNA was higher in all thyroid-disease groups than in controls, but cfDNA did not distinguish malignant from benign disease (*p* = 0.65) or thyroiditis (*p* = 0.29).	NR	No	Relatively small heterogeneous subgroups; marked overlap between disease categories; limited disease specificity.	[115]
Total cfDNA	Postoperative DTC	254 DTC; 95 active structural disease; 159 disease-free	Plasma	Quick-cfDNA Serum & Plasma Kit extraction; Qubit 4 fluorometry	Prediction of postoperative disease status	cfDNA was slightly higher in active structural disease, but the difference was not significant (*p* = 0.122). Effect estimate: NR.	NR	No	Single-center prospective study; postoperative population; single time-point measurement; total cfDNA rather than tumor-specific DNA.	[122]
Tumor-informed cfDNA methylation (ThyMet)	PTC vs. benign thyroid nodules	408 enrolled overall; Phase III: 113 PTC and 88 BTN; validation subset: 47 PTC and 24 BTN	Plasma	Targeted cfDNA methylation sequencing	Preoperative discrimination of PTC from benign nodules	Six-marker ThyMet classifier discriminated PTC from BTN; integration with ultrasound further improved diagnostic performance. Effect estimate: NR (diagnostic-classifier study).	Validation: AUC 0.828; sensitivity 0.750; specificity 0.723. ThyMet-US: AUC 0.923; sensitivity 0.957; specificity 0.708	Internal held-out validation cohort; no external validation	Tumor-associated methylation classifier; not a measure of NET formation; single-study validation; relatively small validation subset; external generalizability remains unconfirmed.	[127]

**Abbreviations:** AUC, area under the receiver operating characteristic curve; BTN, benign thyroid nodule; cfDNA, cell-free DNA; CitH3, citrullinated histone H3; De-DTC, dedifferentiated differentiated thyroid carcinoma; DTC, differentiated thyroid carcinoma; GD, Graves’ disease; GO, Graves’ ophthalmopathy; HT, Hashimoto’s thyroiditis; MNG, multinodular goiter; MPO-DNA, myeloperoxidase-DNA complexes; PTC, papillary thyroid carcinoma; SAA, serum amyloid A; CI, confidence interval; ELISA, enzyme-linked immunosorbent assay; OR, odds ratio. NR, not reported. For other candidate DAMPs discussed in this review, including HSPs and decorin, current thyroid-specific evidence is predominantly tissue-based, bioinformatic, or preclinical, and clinically validated circulating diagnostic-performance data are not currently available. The ThyMet assay is included for diagnostic comparison as a tumor-informed cfDNA methylation approach and should not be interpreted as a marker of NET formation.

## Data Availability

The original contributions presented in this study are included in the article. Further inquiries can be directed to the corresponding author.
